# Verification and characterization of an alternative low density lipoprotein receptor-related protein 1 splice variant

**DOI:** 10.1371/journal.pone.0180354

**Published:** 2017-06-29

**Authors:** Marlen Kolb, Susanne Kurz, Angelika Schäfer, Klaus Huse, Andreas Dietz, Gunnar Wichmann, Gerd Birkenmeier

**Affiliations:** 1 Clinic of Otorhinolaryngology, Head and Neck Surgery, University Hospital Leipzig, Leipzig, Germany; 2 Bio- and Nanotechnology, Fraunhofer Institute for Ceramic Technologies and Systems IKTS, Leipzig, Germany; 3 Rudolf-Schönheimer-Institute of Biochemistry, University of Leipzig, Leipzig, Germany; 4 Leibniz Institute on Aging-Fritz Lipmann Institute (FLI), Jena, Germany; Duke University School of Medicine, UNITED STATES

## Abstract

**Background:**

Low density lipoprotein (LDL) receptor-related protein 1 (LRP1) is a ubiquitously expressed multi-ligand endocytosis receptor implicated in a wide range of signalling, among others in tumour biology. Tumour-associated genomic mutations of the *LRP1* gene are described, but nothing is known about cancer-associated expression of LRP1 splice variants Therefore, the focus of this study was on an annotated truncated LRP1 splice variant (BC072015.1; *NCBI GenBank*), referred to as smLRP1, which was initially identified in prostate and lung carcinoma.

**Methods:**

Using PCR and quantitative PCR, the expression of LRP1 and smLRP1 in different human tissues and tumour cell lines was screened and compared on tumour biopsies of head and neck squamous cell carcinoma (HNSCC). Using a recently developed anti-smLRP1 antibody, the expression of the putative LRP1 protein isoform in tumour cell lines in Western blot and immunofluorescence staining was further investigated.

**Results:**

The alternative transcript smLRP1 is ubiquitously expressed in 12 human cell lines of different origin and 22 tissues which is similar to LRP1. A shift in expression of smLRP1 relative to LRP1 towards smLRP1 was observed in most tumour cell lines compared to healthy tissue. The expression of LRP1 as well as smLRP1 is decreased in HNSCC cell lines in comparison to healthy mucosa. *In vitro* results were checked using primary HNSCC. Furthermore, the expression of the protein isoform smLRP1 (32 kDa) was confirmed in human tumour cell lines.

**Conclusions:**

Similar to LRP1, the truncated splice variant smLRP1 is ubiquitously expressed in healthy human tissues, but altered in tumours pointing to a potential role of smLRP1 in cancer. Comparative results suggest a shift in expression in favour of smLRP1 in tumour cells that warrant further evaluation. The protein isoform is suggested to be secreted.

## Introduction

The low density lipoprotein (LDL) receptor-related protein 1 (LRP1) is an ubiquitously expressed scavenger receptor of the LDL receptor family mediating clearance and recycling of its many structurally and functionally different ligands comprising proteases and proteinases, as well as their inhibitor-complexes, apolipoproteins, growth factors, matrix metalloproteinases, viruses, and bacterial toxins [1,2]. Depending on available ligands and co-receptors, LRP1 also mediates signalling regulating lipid metabolism [3], cell survival, angiogenesis, migration [4], proliferation, as well as cell differentiation [1]. Hence, dysregulation of LRP1 expression is associated with pathogenic states such as atherosclerosis, Alzheimer disease, and tumours [5–7]. Regarding the latter, changes in LRP1 expression, *e*.*g*. due to mutations and gene amplification are associated with increased risk, invasiveness, and poor prognosis [8–13].

**Fig 1:**
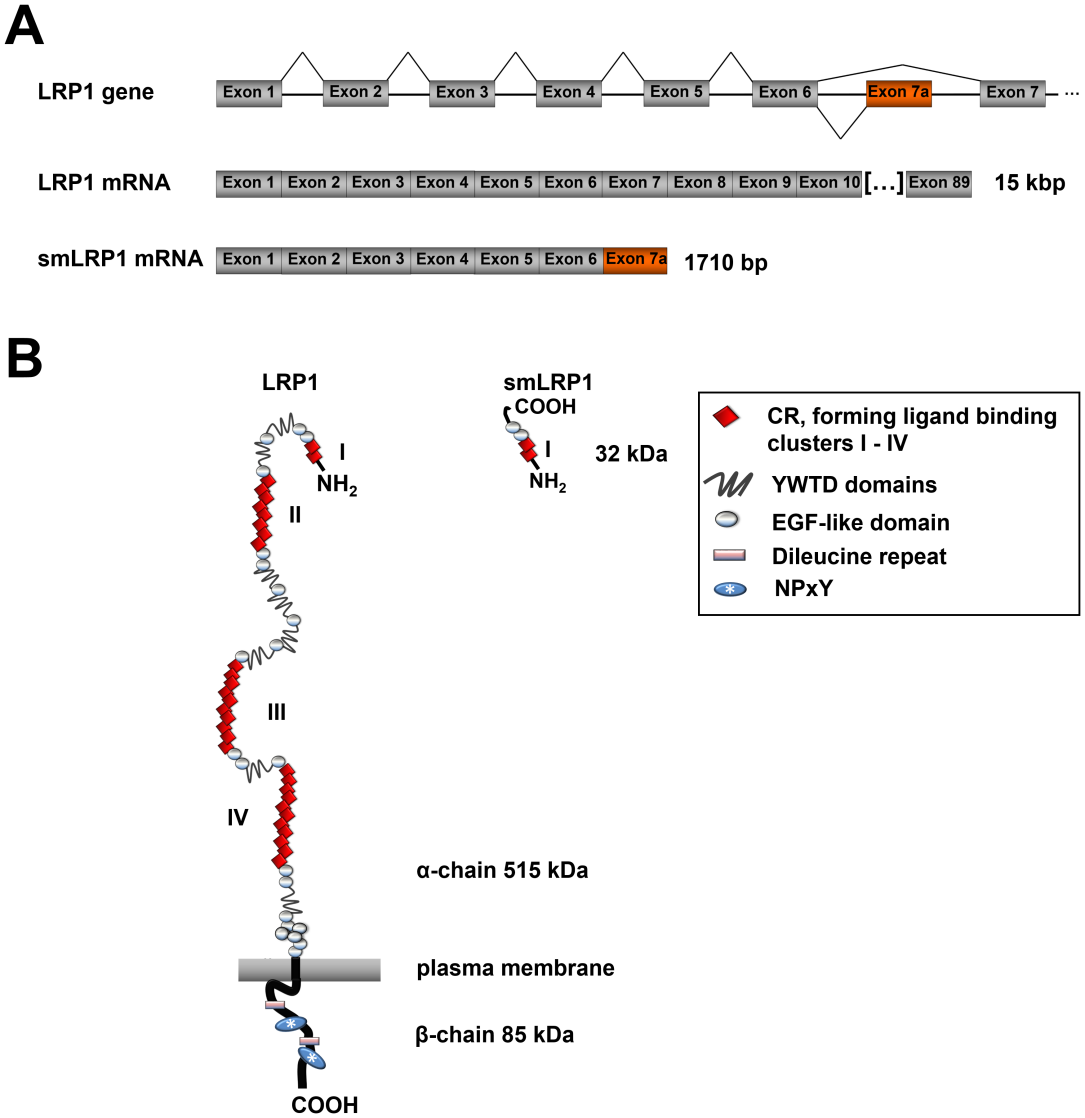
The Low density lipoprotein receptor-related protein 1 and its truncated isoform “smLRP1”. (**A**) The human gene *LRP1* is located on chromosome 12q13-14. Alternative splicing produces the alternative transcript BC072015.1 (NCBI database), called smLRP1, a truncated mRNA comprising 6 protein-coding exons identical to LRP1 and an alternative exon 7 (exon 7a) containing a stop codon and a polyadenylation signal evading nonsense-mediated decay. (**B**) The LRP1 comprises an extracellular α-chain (515 kDa) containing 4 ligand binding clusters and a non-covalently associated transmembrane-spanning β-chain (85 kDa), whereas a molecular mass of 32.4 kDa was calculated for smLRP1. *Abbreviations*: LRP1, lipoprotein receptor-related protein 1; CR, complement like, cysteine-rich sequence repeats; EGF-like, epidermal growth factor-like.

The human *LRP1*, which is localised on chromosome 12q13–14, comprises 89 protein coding exons and further 6 alternative last, non-overlapping exons localised in introns [6,14,15] (Fig 1A). It encodes the largest transmembrane receptor of 4,525 amino acids (aa) structurally organised in an α-chain (515 kDa) and a non-covalently associated β-chain (85 kDa). The former contains 31 complement-like cysteine-rich ligand binding repeats (CR) forming 4 ligand binding clusters consisting of two or more CRs, 22 cysteine-rich epidermal growth factor-like domains (EGF-like) and 8 YWTD domains that are all linked by spacer regions. The β-chain spans the cell membrane followed by an intracellular domain of 100 aa containing two di-leucine motifs and two NPxY motifs. Therefore, LRP1 binds to numerous adapter proteins and regulates signalling [1] (Fig 1B).

To date several truncated splice variants of LRP1 were annotated by the mammalian genome sequencing project in 2002. Though, no further experimental data has been published yet. Alternative splicing describes a frequent event in gene control that expands the proteome encoded by a limited number of genes. Consequently, protein isoforms with altered function, binding properties, stability, and localisation are expressed or protein expression is abrogated due to nonsense-mediated decay of mRNAs [16]. Hence, aberrant splicing is often observed in disease, *e*.*g*. cancer [17,18].

In this study, expression of one of LRP1’s splice variants, referred to as “small LRP1” (smLRP1), that was initially detected in large lung cell and prostate carcinoma [19] was investigated. Besides screening mRNA expression in human tumour cell lines and healthy tissues, a smLRP1-specific monoclonal antibody was developed. For the first time, expression of the potentially encoded truncated protein isoform smLRP1 was confirmed in tumour cell lines. Due to initial screening results, validation of mRNA expression focused on head and neck squamous cell carcinoma (HNSCC). HNSCC cover more than 90% of head and neck tumours, the six-most frequent malignancy in men. It comprises a heterogenic group of tumours derived from neoplasia of the epithelia of the mucosa from the oral cavity, nasopharynx, oropharynx, larynx, paranasal sinus, and salivary gland [20]. Despite improvements in surgery and therapy during the last decades, still more than 50% of the patients die within 5 years [21]. To date, known independent main risk factors are alcohol and tobacco consumption [22] whereas human papillomavirus infection is a further important factor in a subset of HNSCC [23,24]. Although, studies characterising gene expression patterns and mutational states in HNSCC were recently published [25,26], no expression data is available distinguishing between LRP1 and its splice variants.

## Material and methods

### *In silico* sequence analysis

Comparative analyses of human smLRP1 mRNA and protein sequences were run using *Clustal Omega* (http://www.ebi.ac.uk/Tools/msa/clustalo/) and *NCBI Protein BLAST* (https://blast.ncbi.nlm.nih.gov/Blast.cgi?PAGE=Proteins). The prediction of glycosylation sites was done using NetGlycate 1.0 and NetNGlyc Server 1.0 [27,28]; disulphide bonding was predicted using Cystate and ProFunc [29,30]. Protein feature prediction was made by similarity and verified by Raptor X [31], ProFunc server [30], PSORT II [32].

#### Material and chemicals

Roti^®^-Mark, Rotiphorese Gel 30, SDS Ultra-Pure, Tris, NaCl, NaVa, NaF, Phenylmethylsulfonylfluorid (PMSF), Tween 20 and sodium deoxycholate from Carl Roth (Karlsruhe, Germany). Coomassie Brilliant Blue R250, *N*,*N*,*N’*,*N’*-tetramethylethylenediamine (TEMED), Triton X-100, paraformaldehyde, and β-mercaptoethanol from SERVA (Heidelberg, Germany); protease inhibitor mix from Sigma-Aldrich (Taufenkirchen, Germany). The nitrocellulose membrane (0.3 μm) and Ficoll were from GE Healthcare (Little Chalfont, UK); RPMI 1640 medium, Dulbecco’s modified Eagle medium (DMEM) supplemented with 4.5 g glucose/L, Opti-MEM^®^, fetal calf serum (FCS), glutamine (100x), penicillin/streptomycin (100 U penicillin/mL; 100 mg streptomycin/mL) and trypsin/ethylenediaminetetraacetic acid (EDTA), non-essential amino acids, lipofectamine 2000, and HAT supplement were from Life Technologies (Darmstadt, Germany). Phenol red- and riboflavin-free RPMI 1640 was from Bio&Sell (Feucht/Nuremberg, Germany) and human total RNA Master Panel II (Lot 1303454A) was from Clontech Laboratories (Madison, WI, USA); LRP1 (#04–03) and monoclonal anti-human LRP1-α-chain antibody (#02–10) from BioMac (Leipzig, Germany), anti-GAPDH from Cell signaling technologies (Danvers, MA; USA), horseradish peroxidase (HRP)-labelled goat anti-mouse from Dako (Hamburg, Germany), and HRP–labelled anti-rabbit antibody from Jackson ImmunoResearch Lab (West Grove, USA). Anti-FLAG antibody (M2) was from Sigma Aldrich (Steinheim, Germany); goat-anti-mouse Alexa Fluor 488, goat-anti-mouse Cy3 and goat-anti-rabbit Cy3 were from Dianova (Hamburg, Germany). Luminata Western HPR substrate was purchased from Merck-Millipore (Darmstadt, Germany). Primers were from biomer.net (Ulm, Germany), and siRNAs targeting nonsense (#D-001810-10-05) and smLRP1 (sense: 5’-AGGAGAACGAGGUGACACAUU, antisense: 5'-UUGUGUCACCUCGUUCUCCUUU) from Thermo Fisher Scientific Inc. (Waltham, MA, USA) were applied.

### Patient description, study approval

After patient’s written informed consent, biopsies of either primary or recurrent histopathological confirmed HNSCC (ICD-M-8070/3, 8071/3, 8083/3) and mucosa were taken under general anaesthesia by surgeons. The study was approved by the ethic committee of the medical faculty of the Leipzig University (ethic votes No. 201–10–12072010 and No. 202-10-1207210). Samples were stored in TRIzol^™^ until isolation of nucleic acids. Characteristics of HNSCC patients according to criteria of the Union for International Cancer Control (UICC) [33] are listed in Table 1.

**Table 1:** Characterisation of HNSCC patients.

Patient	Age at diagnosis	Gender	Tumor site	UICC
1	70	M	Hypopharynx	III
2	67	M	Larynx	IVA
3	71	F	Oral cavity	IVC
4	59	M	Larynx	IVC
5	60	M	Oropharynx	IVA
6	59	M	Oropharynx	IVA
7	76	M	Oropharynx	IVA
8	75	M	Oropharynx	IVB
9	38	M	Oral cavity	IVA
10	87	F	Oral cavity	III
11	77	M	Larynx	II
12	54	M	Oropharynx	IVA
13	45	F	Nasopharynx	IVB
14	52	M	Hypopharynx	IVA
15	63	M	Larynx	IVC
16	59	M	Oropharynx	IVA

*Abbreviations*: UICC, *Union for International Cancer Control*.

### Cell culture

Human cell lines glioma U87-MG (ATCC^®^ HTB-14^™^), astrocytoma 1321N1 (ATCC LH-1), lung adenocarcinoma A549 (ATCC CCL-185), mamma carcinoma MDA-MB-231 (ATCC HTB-26) and JIMT-1 (DSMZ, ACC 589) were cultured in DMEM, colorectal adenocarcinoma HT-29 (ATCC^®^ HTB-38^™^) in F12 DMEM; all supplemented with 10% FCS, 100 U penicillin, and 100 μg streptomycin/ml. U87-MG were cultured in DMEM as described above and supplemented with 1% non-essential amino acids. Prostate carcinoma PC-3 (ATCC CRL-1435), LnCaP (DSMZ; ACC 256), acute monocytic leukaemia THP-1 (ATCC LH-1), and chronic myelogenous leukaemia K562 (ATCC^®^ HTB-38^™^) were cultured in RPMI supplemented with 10% FCS, 100 U penicillin, and 100 μg streptomycin/ml, whereas HNSCC FaDu (ATCC HTB-43) and HN-5 (Boehringer Ingelheim) were cultured in phenol red- and riboflavin-free RPMI 1640 supplemented with antibiotics. All cell lines were cultured at 37°C/5% CO_2_ in humidified atmosphere (standard conditions).

### Isolation of human peripher blood mononucleic cells

Human peripheral blood mononucleic cells (PBMCs) were isolated according [34] and cultured in RPMI supplemented with 10% FCS, penicillin/streptomycin (100 units/ml and 100 μg/ml) in FCS coated flasks overnight. Media and floating cells were discarded; attached mononucleic cells were harvested for RNA isolation.

### DNA transfection and RNA interference

U87-MG and A549 cells were seeded in 24-well plates. After 24 h, at a cell density of 50–70%, cells were transfected with 500 ng vector pcDNA3.1_Flag-smLRP1 per well using lipofectamine 2000 according to manufacturer’s manual (Life technologies, Darmstadt). Medium was changed after 2 h and cells were cultured for 24 h under standard conditions. For RNA interference, U87-MG cells were seeded and transfected after 24 h at a cell density of 30% in 24-well plates. For transfection, lipofectamine 2000 and 10 nM nonsense control and smLRP1-specific siRNA, respectively, were mixed according to manual and applied onto cells. Transfection media was replaced after 2 h and cells were incubated for 48 h under standard conditions. Then, cells were harvested for RNA and protein extraction. Independent experiments were performed three times.

### RNA extraction and cDNA synthesis

Total RNA of cell lines and PBMCs were isolated using peqGOLD Total RNA isolation kit (Peqlab); extraction from human biopsies was achieved as previously described [25]. RNA integrity was verified by electrophoresis through a 0.8% agarose gel. RNA was reverse transcribed into cDNA using RevertAid First Strand cDNA Synthesis Kit according to the manual (Fermentas by Thermo Fisher Scientific Inc., Waltham; MA, USA).

### Gene expression analysis

For PCR, forward (f) and reverse (r) primer sequences used were as follows: GAPDH (NM_002046.3), f: 5’- AAGGGTCATCATCTCTGCCC—3 and r: 5’- ATGATGTTCTGGAGAGCCCC -3’; (b) LRP1 (NM_002332.2), f: 5- ATCGTGCCGCGAGTATGCCG– 3’ and r: 5’–GTGTGGCGCGTGATGGTGGA -3’, (c) smLRP1 (BC072015.1) f: 5’- CCTGTGCTGTTGATAGCCAA– 3’ and r: 5’–CGTGTTGTGTCACCTCGTTC -3’. For qPCR, forward (f) and reverse (r) primer sequences used were as follows: GAPDH (NM_002046.3) f: 5’-TCAACGGATTTGGTCGTATTGG-3 and r: 5’-GCAACAATATCCACTTTACCAGA -3’; (b) LRP1 (NM_002332.2), f: 5’-TAAAGGGCTTCGTGGATGAG-3’ and r: 5’–GAAGTTGCCTGTCAGCCAGT-3’; (c) smLRP1 (BC072015.1), f: 5’-TAAAGGGCTTCGTGGATGAG-3’ and r: 5’-CGTGTTGTGTCACCTCGTTC-3’; (d) RPL-27 (NM_000988), f: 5’–TCTGGTGGCTGGAATTGACC and r: 5’-CACAGAGTACCTTGTGGGCA-3’.

Target genes were amplified using 0.5 μM of each primer, 100 μM dNTPs, 1 x GoTaq buffer ready to load and 0.025 U/μl GoTaq polymerase (Promega, Madison; WI, USA). GAPDH and LRP1 were amplified in a standard 3-step PCR with 30 cycles; annealing was done at 61°C (GAPDH) and 68°C (LRP1), respectively. For smLRP1, a 2-step protocol was applied with 35 cycles; annealing was done at 60°. PCR products were electrophoresed through a 1.5% agarose gel and visualised under an ultraviolet transilluminator. For quantitative real-time PCR, reactions were done according to manual Platinum^®^ SYBR Green Supermix UDG (Thermo Fisher Scientific Inc.); melt curves were measured during heating from 50°C to 95°C and relative expression of target genes in tumour cell lines compared to healthy tissue was referred to RNA content. For human biopsies, relative gene expression was referred to GAPDH and RPL-27 using Bestkeeper software [35].

### smLRP1 antibody production

The polyclonal anti-smLRP1 antibody was generated in rabbits and the monoclonal in Balb/c mice against the synthesized peptide RRSRKRAQENEVTQHG coupled to hemocyanin dispersed in complete Freund´s adjuvant. Hybridoma cells and monoclonal antibodies were prepared as described earlier according to standard protocol [36].

### Protein extraction, determination of protein content and immunoblotting

Adherent cell lines were treated with 0.04% trypsin/0.03% EDTA. Total protein cell extracts were prepared using RIPA buffer (150 mM NaCl, 50 mM Tris, 1% Triton X-100, 0.5% sodium deoxycholate, 0.1% SDS) supplemented with 2 mM EDTA, protease inhibitor mix (P8340), 1 mM NaVa, 10 mM NaF, and 1 mM PMSF. Protein extracts were onto SDS gels and transferred onto nitrocellulose membranes by blotting. The membranes were blocked and incubated with primary antibody anti-GAPDH and anti-LRP1 according to manual and hybridoma culture supernatant (murine anti-smLRP1, monoclonal), respectively, over night at 4°C. After washing, blots were incubated with HRP-conjugated goat-anti-mouse IgG and HRP-conjugated goat-anti-rabbit IgG antibody for 1 h at room temperature. All antibody dilutions were prepared in TBS-T/0.5% skimmed milk powder according to manuals. Bands were visualized by chemiluminescence.

### Immunofluorescence staining

Cells were fixed with PBS/4% paraformaldehyde for 45 min at 4°C, permeabilized for 15 min at RT using PBS/0.1% Triton-X 100 and blocked for 30 min at 37°C with PBS/10% FCS. For single staining, transfected cells were incubated with primary antibody anti-FLAG (mouse, monoclonal; 1:2,000), anti-LRP1 (mouse, monoclonal; 1:100), and anti-smLRP1 (rabbit, polyclonal; 1:500) for 3 h at 37°C. For co-staining, cells were incubated with anti-FLAG for 1.5 h, washed with PBS and incubated with anti-smLRP1 (rabbit, polyclonal) for 1.5 h at 37°C. After washing, cells were incubated with 0.2 μg 4',6-diamidino-2-phenylindole dihydrochloride (DAPI) in the presence of secondary antibody goat-anti-mouse Alexa Fluor 488 and/or goat-anti-rabbit Cy3 antibody (co-staining) and goat-anti-mouse Cy3 (single staining), respectively, over night at 4°C. Specificity controls were run and pictures taken using immunofluorescence microscope Leica DM IRBE (Wetzlar, Germany).

### Statistical analysis

Differences in gene expression were tested for significance using two-sided, unpaired student’s *t*-test. Wilcoxon signed rank *t*-test was applied for paired mucosa and HNSCC samples. Mann Whitney U test was used for unpaired HNSCC samples (GraphPad Prism Version 5.00 for Windows; San Diego; CA, USA). Two-sided *p*-values are indicated as significant accordingly: *, *p* < 0.05; **, *p* < 0.01, and ***, *p* < 0.001.

## Results

### *In silico* analysis

**Fig 2:**
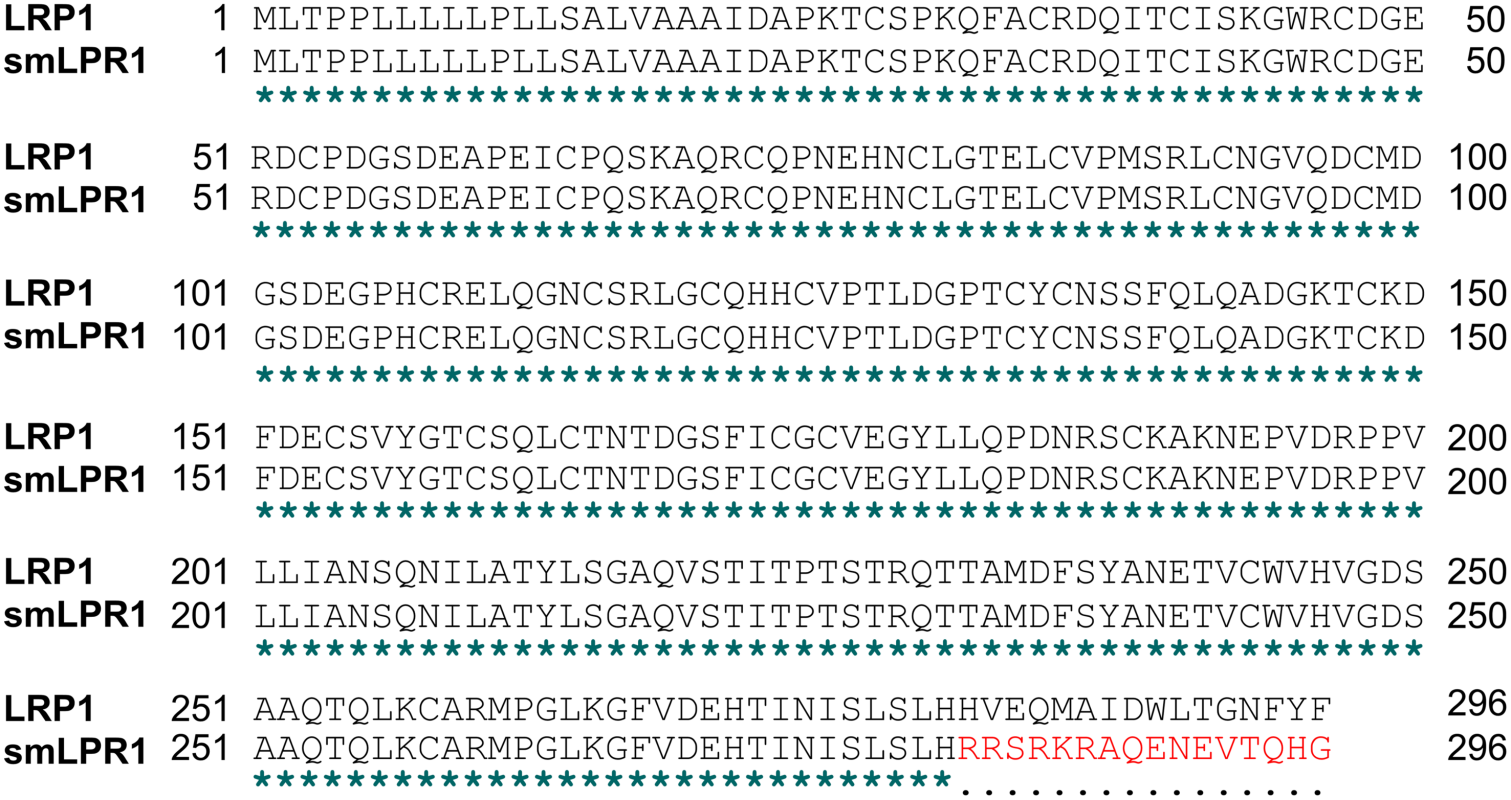
LRP1 and the putative protein isoform smLRP1 share high sequence identity. Sequence alignment of LRP1 (NP_002323.2) and smLRP1 (AAH72015.1) was done using online tool *Clustal Omega* (http://www.ebi.ac.uk/Tools/msa/clustalo/).

A search for smLRP1 sequences in the NCBI database resulted in 2 annotated identical copy DNA clone sequences that were derived from lung and prostate carcinoma (GenBank: BC052593.1 and BC072015.1) and annotated by the mammalian genome sequencing project [19]. The smLRP1 mRNA of 1710 bp comprises 6 protein-coding exons identical to LRP1 and an alternative exon 7 (exon 7a) that contains a stop codon and a polyadenylation signal. Consequently, the splice variant evades nonsense-mediated decay (Fig 1A) and potentially encodes a truncated protein isoform of 296 aa (AAH72015.1) with a calculated molecular mass of 32.2 kDa (Fig 1B). Comparing both sequences, they share an overall identity of 97.5% and 96.6% for mRNA and the potentially encoded protein, respectively (Table 2, Fig 2). Both proteins have a cleavable N-terminal signalling peptide sequence (aa positions 1–20) which is followed by two CR domains [30,31], a hallmark of the LDL family. In both smLRP1 and LRP1, the CR domains are located at position 26–66 and 71–109 forming the ligand binding cluster I. Also identified by similarity, smLRP1 comprises 2 EGF-like domains (regions 112–149 and 150–189) followed by a C-terminal unique aa sequence RRSRKRAQENEVTQHG encoded by the alternative exon 7a absent in LRP1 (Fig 2; Table 3). In total, smLRP1 contains 3 calcium-binding sites by similarity [30,31]. LRP1 comprises 52 potential N-glycosylation sites and 159 disulphide bonds in total [37] (Table 3). Consequently, smLRP1 and LRP1 share 4 potential N-glycosylated aa at position 136, 185, 239, and 274 whereas one putative was additionally identified in smLRP1 at position 114 [27,28]. However, *in silico* analysis yields 11 disulphide bonds for the LRP1-identical sequence of smLRP1 [29,30] (Table 3). Due to intramolecular hydrogen bonds, smLRP1 should form primarily antiparallel β-strands whereas the unique C-terminus is in low order [30,31,38]. The protein isoform smLRP1 comprises neither a transmembrane domain [39] nor motifs for post-translational modifications mediating anchorage in the plasma membrane [32] such as glycosyl-phosphatidyl-inositol, prenylation or N-myristoylation (Table 3). Consequently, smLRP1 is likely to be secreted [32,40,41].

**Table 2:** Comparative sequence analysis of LRP1 and its truncated splice variant smLRP1.

**mRNA**	**LRP1**	**smLRP1**
NCBI GenBank ID	NM_002332.2	BC072015.1*, BC052593.1
mRNA [kbp]	15	1.7
Identity RNA cds		97.5%
**protein**		
Ref Seq	NP_002323.2	AAH72015.1
Amino acids	4,525	296
Molecular Weight [kDa]	600, 515 (α-), 85 (β-chain)	32.4
Identity protein		96.6%
Protein existence	Evidence at protein level	Evidence at transcriptional level
References	Herz *et al*. 1988 [42]	annotation by Strausberg *et al*. 2002 [19]

* *RefSeq used for further analysis*.

*Abbreviations*: cds, coding sequence.

**Table 3:** Protein sequence features of smLRP1.

Features	Position(s)	Length	Description
			**Molecular processing**
Signal sequence	1–20	20	By similarity
Chain	21–296	276	Chain
			**Regions**
Region	26–66	40	CR
Region	71–109	38	CR
Region	112–149	37	EGF-like Domain
Region	150–189	39	EGF-like Domain
Ca^2+^ coordinating	R90, D97, D103,		Ca^2+^ binding
Ca^2+^ coordinating	H107, E110, Q112, G130		Ca^2+^ binding
Ca^2+^ coordinating	D50, D168, T167		Ca^2+^ binding
Disulphide bond	27–40		by similarity
Disulphide bond	34–53		by similarity
Disulphide bond	34–53		by similarity
Disulphide bond	79–98		by similarity
Disulphide bond	82–85		by similarity
Disulphide bond	92–108		by similarity
Disulphide bond	120–133		by similarity
Disulphide bond	135–148		by similarity
Disulphide bond	154–164		by similarity
Disulphide bond	160–173		by similarity
Disulphide bond	175–188		by similarity
glycosylation	114		potential
glycosylation	136		by similarity
glycosylation	185		by similarity
glycosylation	239		by similarity
glycosylation	274		by similarity

*Abbreviations*: CR complement-like cysteine-rich sequence repeats, EGF epidermal growth factor.

### LRP1 and smLRP1 are differentially expressed in human tumour cell lines and corresponding healthy tissues

**Fig 3:**
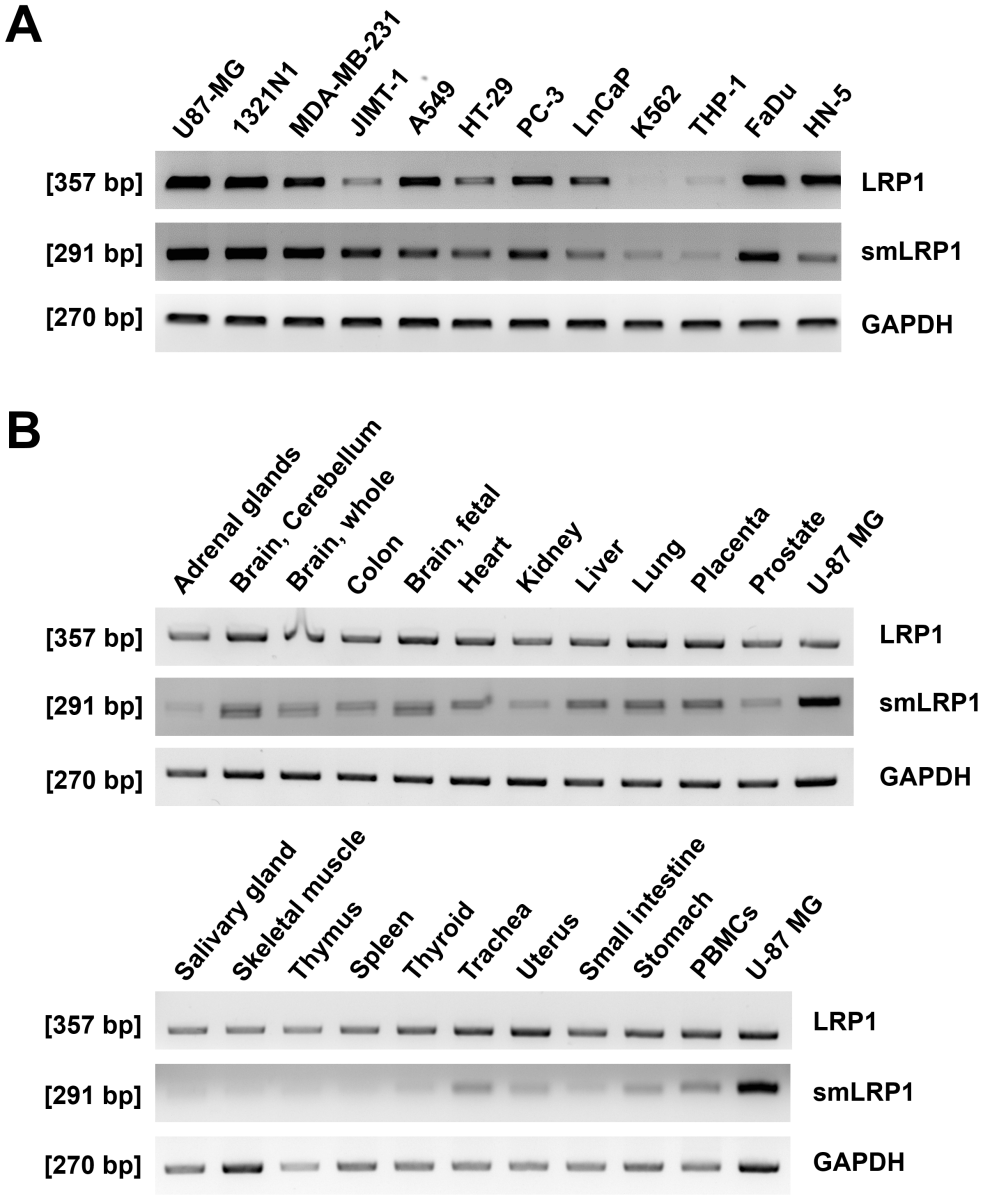
LRP1 and its truncated splice variant smLRP1 are ubiquitously expressed in human tissues and tumour cell lines. (**A**) Human tumour cell lines, (**B**) healthy tissues and PBMCs were screened for LRP1 and smLRP1 mRNA expression in PCR using GAPDH as control.

**Fig 4:**
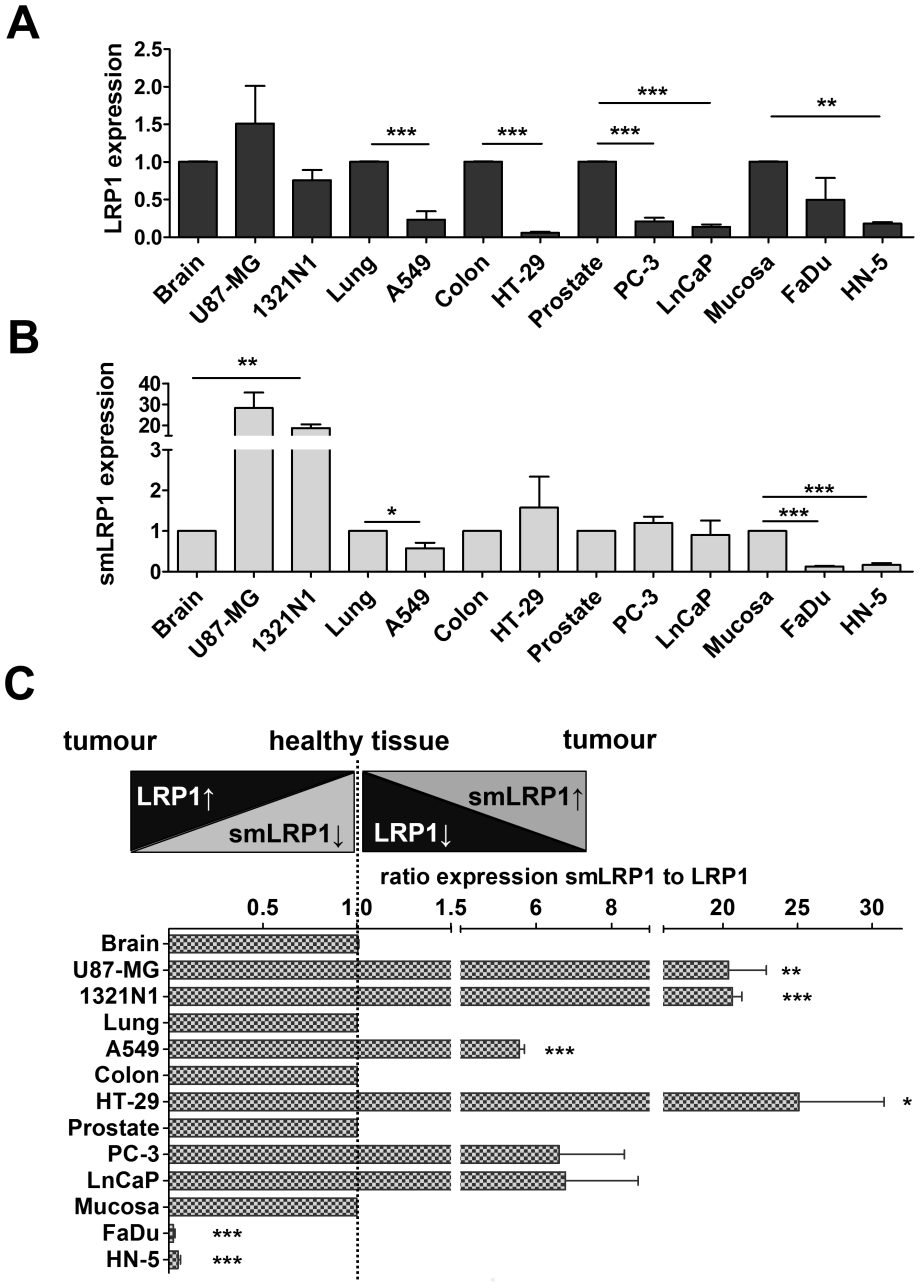
LRP1 and smLRP1 are differentially expressed in tumour cell lines and healthy tissues. (**A**) Quantitative Real-time PCR showed a shift in relative expression of LRP1 and (**B**) smLRP1 in tumour cell lines. (**C**) Comparing expression of smLRP1 relative to LRP1 (ratio smLRP1/LRP1) in 5 human tissues and their corresponding tumour cell lines indicated a shift in expression in favour of smLRP1 in most tumour cell lines. Results are presented as mean±SEM of 3–16 biological replicates. Significant results are highlighted accordingly: *, *p*<0.05; **, *p*<0.01, and ***, *p*<0.001.

As smLRP1 was first detected in lung and prostate carcinoma [19], 12 human tumour cell lines of different origin were screened for expression of smLRP1 and LRP1 (Fig 3A). LRP1 mRNA was expressed in all screened cell lines. Using smLRP1 specific primers, expression of smLRP1 mRNA was confirmed in non-small lung cell carcinoma A549, prostate carcinoma, PC–3 and LNCaP as well as further cell lines derived from astrocytoma (1321N1), glioblastoma (U87–MG), mamma carcinoma (MDA-MB-231 and JIMT-1), colon carcinoma (HT-29), leukaemia (THP-1 and K562), and HNSCC of the hypopharynx (FaDu) and tongue (HN-5). Subsequently, expression was checked in a panel of healthy human tissues and PBMCs. Cell line U87-MG served as positive expression control for smLRP1 (Fig 3B). PCR analysis showed ubiquitous expression of both LRP1 and smLRP1 in healthy tissue. LRP1 was abundantly expressed in foetal and adult brain, placenta, lung, liver, and colon as described in literature [43]. Generally, smLRP1 expression pattern among tissues is similar to LRP1 (Fig 3B). Thus, expression of smLRP1 and LRP1 was compared between 5 healthy tissues and corresponding cell lines using qPCR (Fig 4A and 4B). Relative LRP1 expression is slightly increased in U87-MG (n = 3) and decreased in A549 (n = 5; *p*<0.001), HT-29 (n = 4; *p*<0.001), PC-3 (n = 4; *p*<0.001), and LnCaP (n = 3; *p*<0.001). Similar to LRP1, expression of smLRP1 is increased in both U87-MG and 1321N1 (*p*<0.01) compared to brain tissue and decreased in A549 (p<0.05) compared to lung. Comparing relative expression in FaDu (n = 3) and HN-5 (n = 3) to mucosa (N = 16) showed decreased expression for both LRP1 (HN5, *p*<0.001) and smLRP1 (FaDu, *p*<0.001; HN5, *p*<0.001; Fig 4A and 4B). Generally, there is a shift in expression of smLRP1 relative to LRP1 between tissues and tumour cells (Fig 4C). Normalising smLRP1 expression to LRP1 and referring its ratio in tumour cell lines to that of corresponding healthy tissues (ratio smLRP1/LRP1) showed increased expression level of splice variant smLRP1 relative to LRP1 in U87-MG (*p*<0.01), 1321N1 (*p*<0.001), A549 (*p*<0.001), and HT-29 (p<0.05) compared to corresponding healthy human tissue. In contrast, the ratio smLRP1/LRP1 was decreased in FaDu (*p*<0.001) and HN5 (*p*<0.001; Fig 4C).

**Fig 5:**
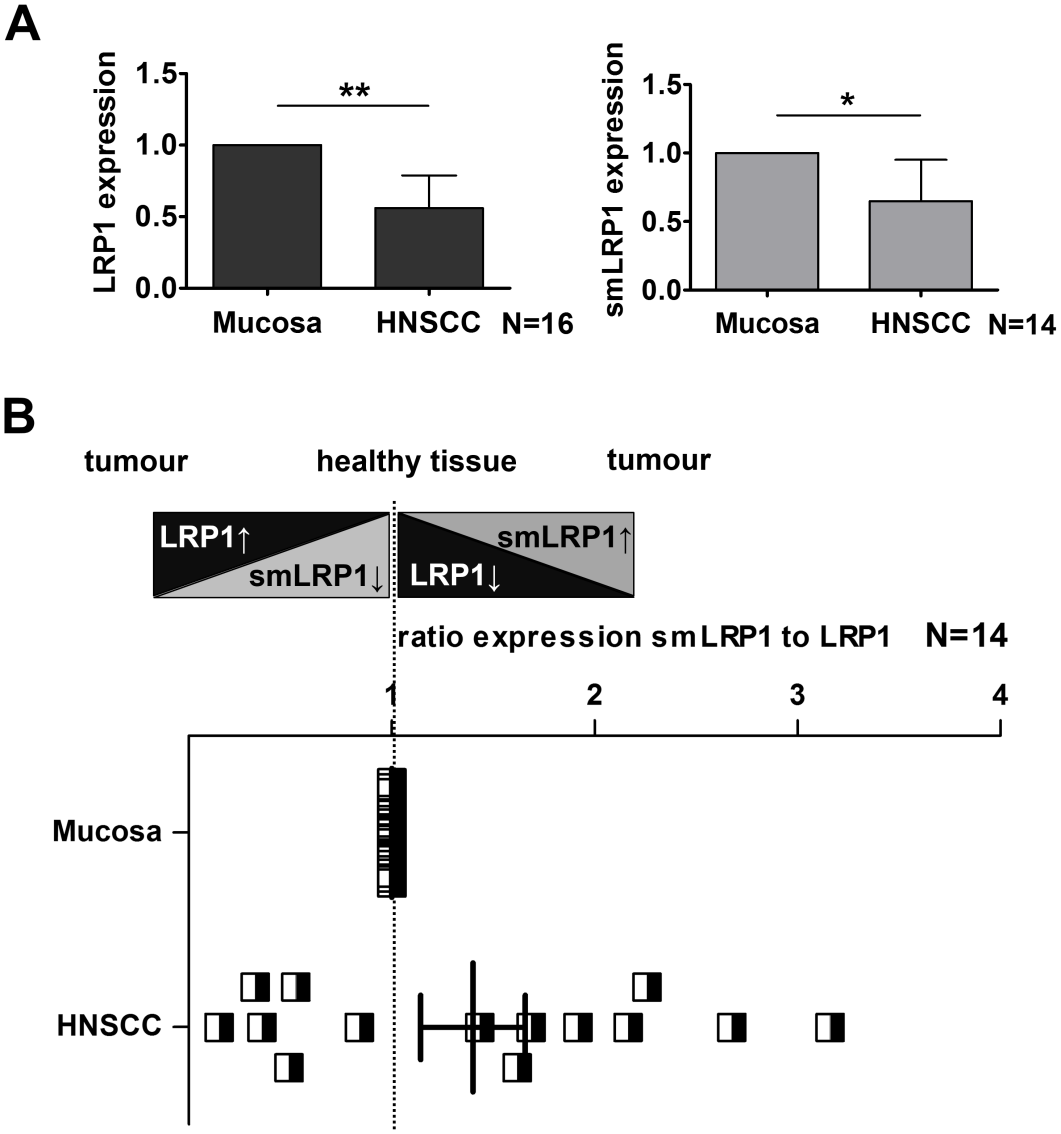
LRP1 and smLRP1 mRNA expression is altered in primary HNSCC compared to mucosa. (**A**) *In vitro* results for HNSCC cell lines were validated by screening paired mucosa and HNSCC specimen (N = 16) for smLRP1 and LRP1 showing decreased mRNA expression of LRP1 and smLRP1. Relative gene expression was referred to GAPDH and RPL27. **(B)** The ratio smLRP1/LRP1 in HNSCC was compared to the corresponding mucosa. Results are presented as mean±SEM of 14–16 biological replicates. Significant results are highlighted accordingly: *, *p*<0.05; **, *p*<0.01, and ***, *p*<0.001.

Due to contrary results in HNSCC cell lines compared to others, our *in vitro* results underwent verification by analysing expression in paired HNSCC and mucosa biopsies from 16 HNSCC patients (Table 1) with a median age of 61 years at diagnosis. The HNSCC samples analysed comprised 6.2% early stage (1/16, UICC II) and 93.8% advanced stage tumours (UICC III-IV). The results confirmed decreased expression of smLRP1 (N = 14, *p*<0.05) and LRP1 mRNA (N = 16; *p*<0.01) in HNSCC compared to mucosa (Fig 5A). However, comparing the ratio smLRP1/LRP1 showed high variability among patients in general, whereas the mean ratio of smLRP1/LRP1 was with 1.402±0.258 slightly increased in HNSCC samples in contrast to initial *in vitro* results (*p* = 0.144; Fig 5B). Interestingly, those patients still alive had a higher ratio smLRP1/LRP1 (mean 1.897±0.327, N = 7) than those deceased (mean 0.9071±0.3177, N = 7, *p* = 0.073).

### Verification of the encoded protein isoform smLRP1 in human tumour cells

**Fig 6:**
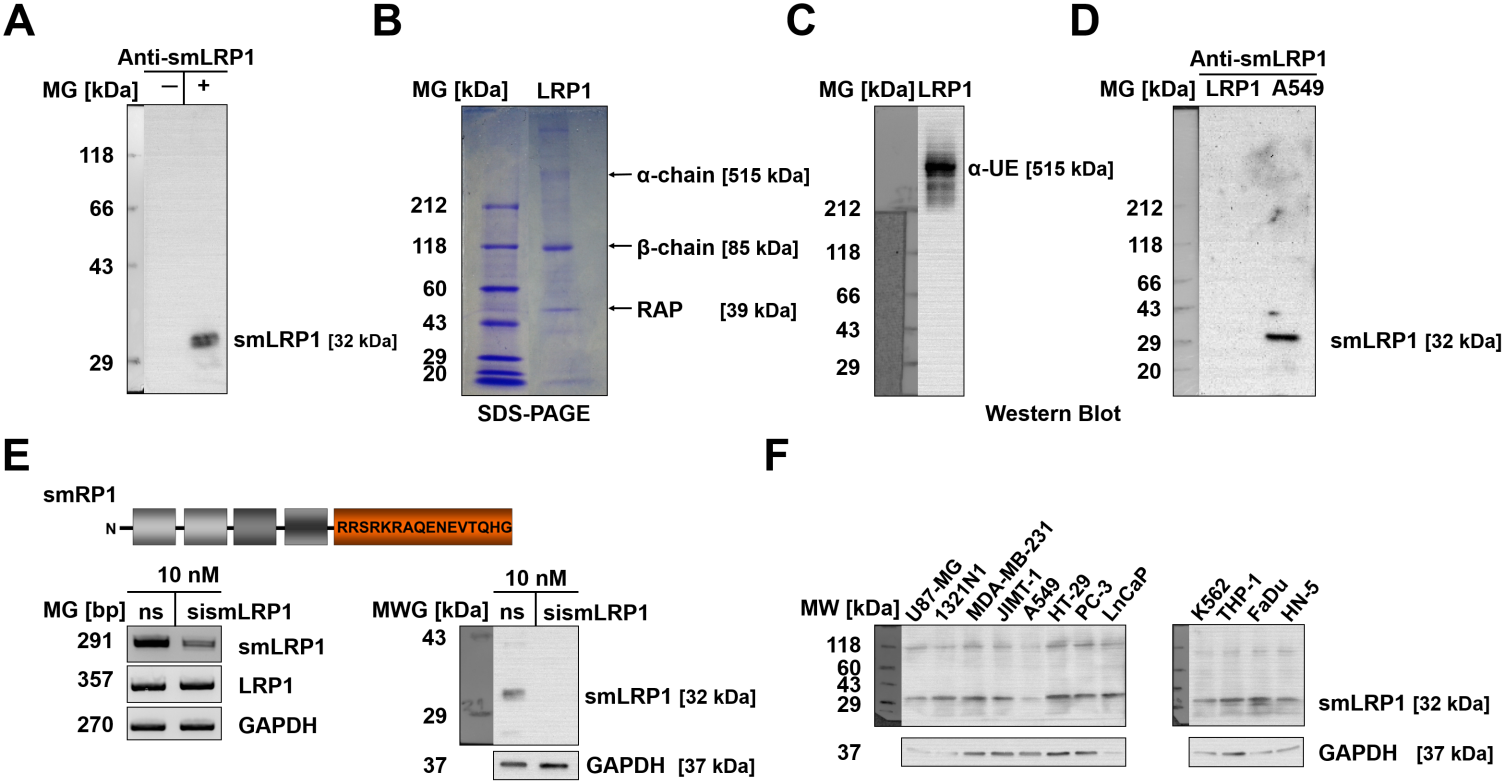
The putatively encoded protein isoform smLRP1 is expressed in human tumour cell lines. Tumour cell lysates (40 μg) and purified LRP1 (1 μg) were subjected to SDS-PAGE under non-reducing conditions and Western blotted using monoclonal murine anti-smLRP1 and anti-LRP1 antibodies targeting the smLRP1 C-terminus (32 kDa) and LRP1 α-chain (515 kDa), respectively. GADPH expression served as control. (**A**) In the absence of secondary anti-mouse antibody (1:5,000), no smLRP1 protein band (32 kDa) could be detected in A459. (**B**) LRP1 comprises an α- (515 kDa) and β-chain and is co-purified with RAP. (**C**) Probing purified LRP1 with anti-LRP1 detected the α-chain with a molecular weight of 515 kDa. (**D**) Contrary, probing both LRP1 and A549 lysate with anti-smLRP1 no binding to the LRP1 α-chain was observed, whereas a smLRP1 protein band (32 kDa) was detected in A549. (**E**) RNAi-mediated transient knockdown of smLRP1 mRNA expression in U87-MG for 48 h resulted in decreased smLRP1 protein expression compared to nonsense control (ns; N = 3). **F**) The smLRP1 protein (32 kDa) was detected in 12 different human tumour cell lines. *Abbreviations*: LRP1, low density lipoprotein receptor-related protein 1; RAP, receptor associated protein; ns, nonsense control.

In order to verify expression of the putatively encoded protein isoform smLRP1, a monoclonal murine antibody targeting the aa sequence RRSRKRAQENEVTQHG encoded by the alternative exon 7a was generated. The specificity of anti-smLRP1 was verified in Western blot (Fig 6). Therefore, whole cell lysates of A549 and U87-MG as well as purified LRP1 (1 μg) were separated in SDS-PAGE under non-reducing conditions and blotted onto a nitrocellulose membrane. Probing A549 cells with anti-smLRP1 showed a 32 kDa protein band correlating with the calculated molecular weight of smLRP1. No such band was observed in absence of secondary antibody (Fig 6A). Moreover, probing purified LRP1 comprising of two subunits (515 kDa and 85kDa, Fig 6B) with anti-LRP1 detected the α- chain at 515 kDa (Fig 6C). In contrast, anti-smLRP1 did not bind to LRP1 α- chain, whereas the antibody detected a protein band (32 kDa) in A549 lysate (Fig 6D). A negative expression control was established by providing a RNA interference-mediated transient knockdown *in vitro*. Incubating U87-MG with 10 nM siRNA for 48 h yielded the expected decrease of the smLRP1 mRNA expression compared to the nonsense control. Analogously, the protein expression was decreased after 48 h compared to control (Fig 6E). Finally, 12 human tumour cell lines were shown to express the truncated protein isoform smLRP1 (32 kDa; Fig 6F).

**Fig 7:**
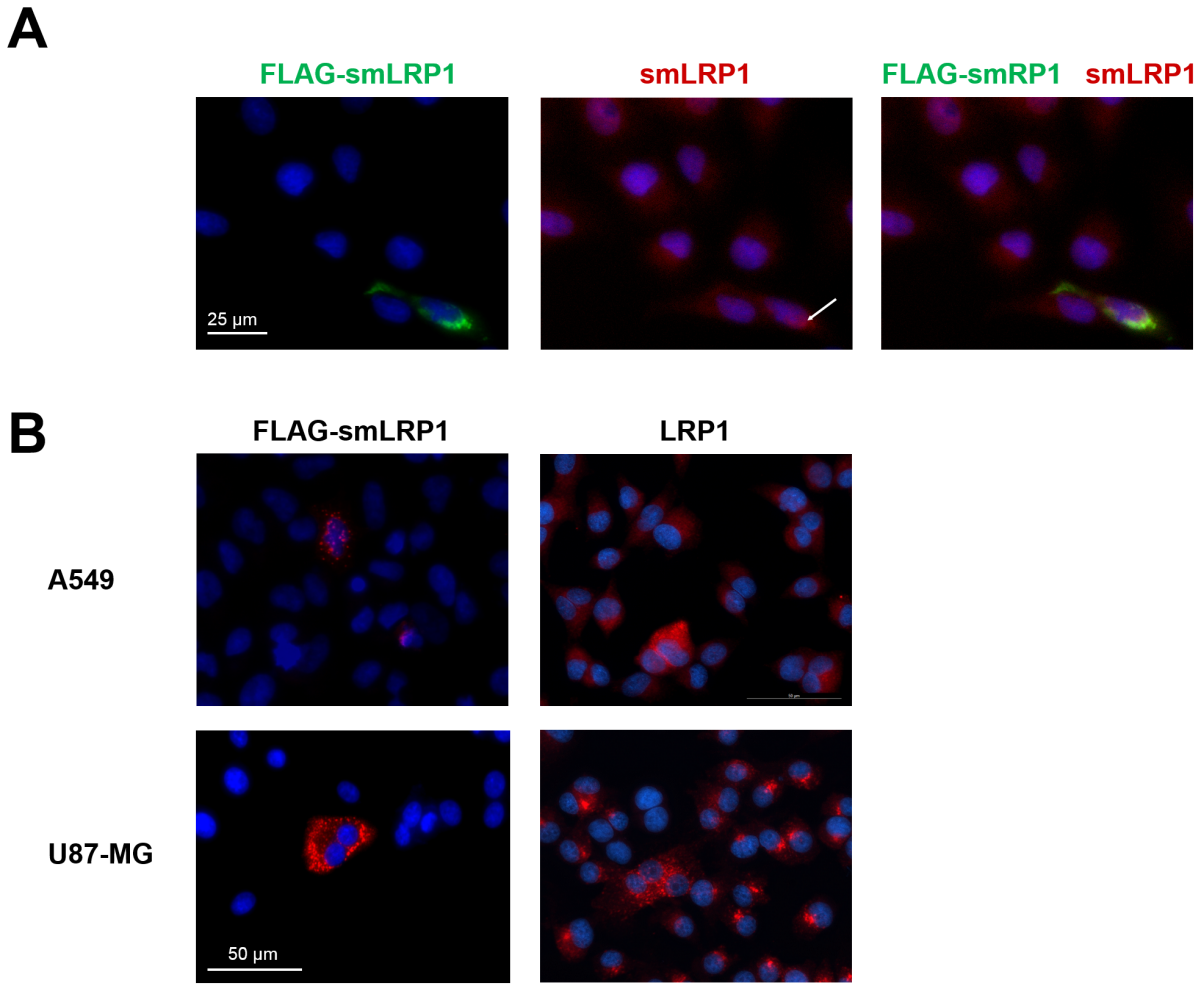
LRP1 and protein isoform smLRP1 are expressed in tumour cells. Tumour cells were fixated with 4% PFA and permeabilized with Triton X-100. (**A**) Co-staining of native smLRP1 using polyclonal anti-smLRP1 (rabbit) and Flag-smLRP1 after transfection with pcDNA3.1_Flag-smLRP1 vector showed concordant localisation of the native and Flag-smLRP1 protein in A549. Staining of (**B**) native LRP1 α-chain and over-expressed Flag-smLRP1 targeting the N-terminal Flag-tag in A549 and U87-MG showed localisation in vesicles in the cytoplasm along the plasma membrane.

Transfecting tumour cells with smLRP1 fused to a N-terminal Flag-tag was followed by co-immunostaining against the Flag-tag of overexpressed Flag-smLRP1 and native smLRP1 using a polyclonal antibody (rabbit). Results showed concordant localisation of both native and Flag-smLRP1 in A549 cells as both are detected by the polyclonal antibody targeting the unique C-terminus of smLRP1 (Fig 7A). With respect to cellular localization, staining LRP1 in A549 and U87-MG after permeabilization indicated its localisation in the plasma membrane in coated pits and in vesicles located among others along the plasma membrane. Transfecting both cell lines with Flag-smLRP1 and staining against the Flag-tag indicated the localisation of Flag-smLRP1 in the cytoplasm, potentially in vesicles, similar to LRP1, whereas no indication of its presence in the plasma membrane was found (Fig 7B).

## Discussion

Our study reveals that expression of the multi-ligand endocytosis receptor LRP1 is ubiquitously accompanied by expression of its truncated splice variant smLRP1 (BC072015.1, Fig 2A and 2B) in all samples tested. To date, the existence of the encoded protein isoform was only predicted. In this study, expression of the *in silico*-predicted smLRP1 protein was confirmed in tumour cells for the first time (AAH72015.1, Fig 5). The scavenger receptor LRP1 is implicated in several signalling pathways due to its wide range of ligands. Thus, changes in LRP1 expression are associated with several pathological states [1]. Concerning tumour biology LRP1 is controversially discussed, since it promotes and inhibits tumour development and progression depending on expression status [7]. The tissue and cancer related expression of LRP1 and its alternative splice variant smLRP1 might add a new aspect to this controversy.

Based on *in silico* analysis, the truncated smLRP1 encodes a protein isoform of 296 aa with an overall identity of 96.6% and a similar protein structure as LRP1 (Table 2). Similar to LRP1, it contains a secretion signal sequence and the first CR domains forming ligand binding cluster I in LRP1, followed by two EGF like domains and a unique C-terminus of 16 aa different from LRP1. The smLRP1 protein structure is likely to be stabilised by 11 intra-protein disulphide bonds and contains 5 potential N-glycosylation sites (Table 3, Fig 2). Hence, the isoform is potentially able to bind LRP1 ligands. In 1999, Neels *et al*. already showed that methylamine-activated α_2_-macroglobuline, apolipoprotein E, lipoprotein lipase, the light chain of blood coagulation factor VIII, lactoferrin, tissue factor pathway inhibitor, tissue-type plasminogen-activator (tPA), plasminogen activator inhibitor-1, tPA-plasminogen activator inhibitor-1 complex, and pro-urokinase-type plasminogen activator do not bind to cluster I [44] using soluble recombinant receptor-fragments of the ligand binding cluster I-IV. Similarly, the chaperon, receptor-associated protein (RAP), only bound to overexpressed cluster constructs II, III, and IV [45,46]. The recombinant cluster I construct was secreted due to presence of the N-terminal signal sequence, whereas the overexpressed cluster II–IV constructs showed little to no secretion. Thus, it seems reasonable to assume that smLRP1 can also be secreted by cells.

So far, alternative splicing is described among others for the transmembrane fibroblast growth factor receptor resulting in expression of its soluble isoform FGFR3 [47]. As for LRP1, it is only known that the LRP1 protein itself can be further processed releasing a soluble LRP1 form. Shedding increases during ageing and is dysregulated in disease wherefore it is abundantly present in brain tissue and cerebrospinal fluid as well as in serum and plasma [48–50]. The released extracellular part of LRP1 (515 and 55 kDa) [51] is still able to bind ligands. Hence, secreted smLRP1 may function as a competitive binding partner for some of LRP1’s ligands in the extracellular space.

Our study showed decreased expression of LRP1 in cell lines derived from non-small lung, prostate and colon carcinoma, all consistent with results described in literature [13,52,53]. The here first described ubiquitous expression of smLRP1 in tumour cell lines of different origins as well as human tissues (Fig 3A and 3B) extents our knowledge about LRP1. Comparing tumour cells and corresponding healthy tissues showed differences in expression of smLRP1 and LRP1 (Fig 4A and 4B). LRP1 expression was decreased in cell lines derived from non-small lung, prostate, and colon carcinoma as described in literature [13,52,53]. Variations in LRP1 expression might be due to varying cell density at time of harvesting the cells [54]. Interestingly, the expression ratio of smLRP1 relative to LRP1 was found to shift in tumour cell lines of brain, lung, colon, and prostate tumour towards higher smLRP1 levels and decreased in HNSCC cell (Fig 4C). The initial results in HNSCC cell lines that showed contrary results compared to cell lines of other entities were validated in 16 primary HNSCC samples. To date, there is only little data on LRP1 in HNSCC. Martínez-Ledesma *et al*. developed a multi network clinical association algorithm to predict clinical outcome in several tumour entities including HNSCC, based on TCGA data. One of the included 22 genes, LRP1, was scarcely mutated in HNSCC (6.2%, 17/276) and there were almost no gains (0.7%, 2/276) and losses of copy numbers [55]. However, previous publications did not distinguish between LRP1 and its splice variants [55,26,25]. Analysing paired biopsies of HNSCC patients confirmed a decreased expression of smLRP1 and LRP1 in HNSCC compared to mucosa (Fig 5A). Nevertheless, smLRP1 expression levels relative to LRP1 highly deviated in HNSCC compared to corresponding mucosa (Fig 5B). The latter is not unexpected since HNSCC describes a very heterogenic group of cancers. Accordingly, we chose a representative patient selection with a median age of 61, primarily male (81.2%) and with an advanced tumour (81.2%), primarily of the oropharynx and larynx (37.5% and 25%; Table 2). Regarding the heterogeneous results of ratio smLRP1/LRP1, different stage and origin of the tumours might contribute to varying results. Summarising, the results suggest a shift of the ratio smLRP1/LRP1 in primary HNSCC. Since expression was analysed retrospectively comprising a limited number of patients, further investigations are necessary.

Expression of the putative smLRP1 protein isoform was verified using a newly developed monoclonal antibody targeting the C-terminal 16 aa peptide sequence encoded by the unique alternative exon 7a due to high sequence identity (96.6%, Table 2, Fig 2). As the *LRP1* gene is phylogenetically highly conserved, the epitope sequence was further investigated for absence of homologues in mice. The developed monoclonal antibody detects a 32 kDa protein under non-reducing conditions in human tumour cell lines matching the calculated molecular weight of smLRP1. As expected, the anti-smLRP1 antibody did not bind to human LRP1 in Western blot allowing specific detection of the smLRP1 isoform. The specificity was further confirmed by RNAi-mediated knockdown of smLRP1 expression *in vitro* resulting in reduced presence of the 32 kDa protein. Finally, it was shown that the protein isoform smLRP1 was expressed in all tumour cell lines of different origin (Fig 6). Localisation in the cell was studied targeting an overexpressed recombinant flag-smLRP1 protein. Immunofluorescence staining of tumour cells showed concordant localisation of Flag-smLRP1 and the endogenous smLRP1. The former was present in vesicles similar to LRP1 which supports the *in silico* results (Fig 7). However, transfection efficiency was unexpectedly low independent of the method of transfection and the cell line tested. Since results were validated in different cell lines, low transfection efficacy might be due to N-terminal position of the flag-tag that allowed parallel staining against the flag-tag and the unique C-terminal sequence of the recombinant protein.

Considering that expression of smLRP1 shifts in a tumour-entity specific manner suggests that smLRP1 might play a role in tumour biology. Alternative splicing is aberrant in tumours and so far, cancer-specific splicing was reported for several entities, among others in prostate, colon, and bladder tumours [18]. Hence, the presence of an alternative splice variant and its encoded protein isoform, even without knowledge of its function, could provide an useful biomarker [56]. Summarising, it was shown for the first time that the splice variant smLRP1 is ubiquitously expressed in human tissues. Moreover, the expression of the predicted protein isoform was experimentally confirmed in cultured tumour cell lines. However, it remains to be clarified if smLRP1 is secreted as indicated by *in silico* analysis. Hence, further investigations are required in order to explore the function of smLRP1 and its potential as a tumour marker.
